# Saving mothers and newborns in communities: strengthening community midwives to provide high quality essential newborn and maternal care in Baluchistan, Pakistan in a financially sustainable manner

**DOI:** 10.1186/1471-2393-14-131

**Published:** 2014-04-06

**Authors:** Zubia Mumtaz, Andrea Cutherell, Afshan Bhatti

**Affiliations:** 1 School of Public Health, University of Alberta, 3-309 Edmonton Clinic Health Academy, 11405 – 87 Ave, Edmonton, AB T6G 1C9, Canada; 2 Mercycorps Pakistan, 152 Margalla Road, Islamabad, Pakistan; 3 Real Medicine Foundation, Pakistan, 70-A, Nazimuddin Road, F-7/4, Islamabad, Pakistan

## Abstract

**Background:**

To address it's persistently high maternal mortality rate of 276/100,000 live births, the government of Pakistan created a new cadre of community based midwives (CMW). One expectation is that CMWs will improve access to maternal health services for underserved women. Recent research shows the CMWs have largely failed to establish midwifery practices, because CMW's lack of skills, both clinical and entrepreneurial and funds necessary to develop their practice infrastructure and logistics. Communities also lack trust in their competence to conduct safe births. To address these issues, the *Saving Mothers and Newborn* (SMNC) intervention will implement three key elements to support the CMWs to establish their private practices: (1) upgrade CMW clinical skills (2) provide business-skills training and small loans (3) generate demand for CMW services using cellular phone SMS technology and existing women’s support groups.

**Methods/Design:**

This 3-year project aims to investigate whether CMWs enrolled in this initiative are providing the essential maternal and newborn health care to women and children living in districts of Quetta, and Gwadar in a financially self-sustaining manner. Specifically the research will use quasi-experimental impact assessment to document whether the SMNC initiative is having an impact on CMW services uptake, financial analysis to assess if the initiative enabled CMWs to develop financially self-sustainable practices and observation methods to assess the quality of care the CMWs are providing.

**Discussion:**

A key element of the SMNC initiative - the provision of business skills training and loans to establish private practices - is an innovative initiative in Pakistan and little is known about its effectiveness. This research will provide emperic evidence of the effectiveness of the intervention as well as contribute to the body of evidence around potential solutions to improve sustainable coverage of high impact Maternal, Neonatal and Child Health interventions in vulnerable populations living in remote rural areas.

## Background

### Context

Pakistan is one of the six countries that contributes to over two-thirds of all maternal deaths worldwide [1]. Within Pakistan, the maternal mortality ratio is highest in the province of Baluchistan (785 per 100,000 live births compared to a national average 276). Here 81% of women deliver at home; 72 percent of them are assisted by a traditional birth attendant. Nationwide, the under-5 mortality is 94 per 1,000 live births, 57 percent of these occur during the first month of life [2].

A large body of evidence suggests high levels of skilled birth attendance is a pre-requisite to the reduction of maternal mortality. The implementation of community midwifery was a major contributor to the decline in maternal and neonatal mortality in Sweden during the late 19th century and in Sri-Lanka and Thailand in the twentieth century [3]. More recently, Bangladesh has shown vast improvements in maternal mortality ratios due to the emphasis on pro-poor strategies like providing free community-based skilled birth attendance [3-5].

Drawing on these experiences, the government of Pakistan (GOP) introduced a new cadre of village-based skilled birth attendants-the community midwife (CMW). With the objective of providing skilled birth attendance to women living in remote areas, the program aimed to deploy 12,000 CMWs over a five year period between 2007 and 2012 [5]. In order to ensure the CMWs practice in rural areas, women were selected from their rural home communities, and deployed back to the same communities after completing an 18-month midwifery course. The training was designed to provide extensive clinical practice including conducting at least 25 normal deliveries as well as primary health care training, particularly for neonatal childhood illnesses. One CMW is expected to cater to a population of approximately 5,000 individuals (total population). The MOH supports these qualified and registered CMWs in establishing their own home-based, private practices in the communities by providing a small stipend for the first three years after graduation.

Recent research, however, suggests that these CMWs have largely failed to establish their midwifery practices in many parts of Pakistan. In Punjab, four years after deployment of the first batch of midwives, 12.7% of deliveries in district Jhelum and 3.4% in district Layyah were attended by a CMW. These results echo the findings of an earlier evaluation in 2010 in Punjab that found 43% of the CMWs had not performed a delivery in the previous three months [6]. Some issues that underlie this poor performance are:

1) Poor training: A number of evaluations have documented the inadequacy of the CMW training program [7,8]. Consequently, less than one third of the CMWS can list the three signs of danger in pregnancy and less than a half can list three signs of danger during childbirth. This lack of competence translates into a lack of confidence that is sensed by the community. Women, both young and old, cite their lack of trust in the CMWs’ abilities to conduct a safe birth as a key reason for not seeking their care. In more remote districts where traditional birth attendants (*dais*) are still the dominant providers, the CMWs are understood to be 'doctors' who should be consulted in the event of a birth complication only. The CMWs however, fail to meet such lofty expectations [6].

2) The CMWs demonstrate a distinct lack of interest in pursuing midwifery practice, primarily because they are unable to meet the professional demands of midwifery practice in remote rural areas. Most are young women and gender norms of rural Pakistani society preclude their travel alone to homes located at significant distances. In Punjab, during day time CMWs require chaperones, preferably an older woman, and at night at least two men in addition to a woman are needed. This greatly increases the opportunity costs of a CMW's practice [6].

3) Income from patient remuneration has remained insufficient for establishment of self-sustaining practices. The CMWs lack business skills necessary to establish private practices. For example, they charge fees that are unaffordable for the majority of patients, which deters women from seeking their care [6].

These issues in establishing midwifery practices are not unique to Pakistan. Indonesia has had similar experiences when it established a community midwife program in 1986 in which over 67,000 CMWs were trained and deployed, one for each village. As in Pakistan, these midwives were not to be a free service. The government gave them 3-year salary contracts, during which time they were expected to develop their private practices [9]. The majority of the CMWs, however, failed to establish their practices. The reasons included questionable midwifery skills, geographic clustering around existing health facilities and providers who created formidable competition, and a large social distance between the providers and patients [10]. Upon expiry of their contracts, the midwives began abandoning their posts in the public sector for lucrative positions in the private sector in urban areas [11,12]. Consequently, maternal mortality in Indonesia has stagnated at 220/100,000 live births [11].

To prevent a similar loss of one of Pakistan’s potentially greatest resources to address maternal and neonatal mortality, the government of Baluchistan and Mercy Corps seek to support this dormant workforce by addressing the four interlinked barriers CMWs face in the provision of maternal and neonatal health care. These include:

1. Developing and conducting a 4 week clinical refresher course and providing ongoing joint supportive supervision throughout the program to ensure CMWs provide high quality care. As a first step, Mercy Corps, in partnership with the Baluchistan Department of Health, will select CMWs who meet the selection criteria from the pool of CMWs trained and deployed by the Baluchistan Maternal and Neonatal Child Health program (See Table 1 for the selection criteria). In the first year, 40 CMWs will be recruited. In year 2, 50 CMWs will be recruited after they are traced out by visiting each and every village in a geographically expanding scope. In the first phase, the recruited CMWs will be provided a four-week refresher training, with technical assistance from the Pakistan Nursing Council (PNC), in district midwifery schools. Mercy Corps will facilitate registration with the PNC for those not already registered.

2. Providing business skills training and linking CMWs with microloans, thereby creating a model that provides an incentive to CMWs to pursue a long-term midwifery practice in marginalized communities (with high quality). Mercy Corps will also provide CMWs with one week business skills training. This training, to be paid for by SMNC, will include helping CMWs develop a business plan for their home-based clinics, develop a loan repayment plan and a mechanism to track contributions and use of the *Mamta* Fund for emergency transport.

Financial assistance will be extended through a microfinance institute (MFI), *Tameer Microfinance Bank*. The MFI will provide loans up to $745 (for basic renovations, repairs, furniture, and an initial supply of medicine) by using Telenor’s *EasyPaisa* service through mobile phones. The use of *EasyPaisa* will ensure that the CMWs can easily access the loans without having to travel to formal banks, which are often located far away from their homes. The CMWs can access the full amount or portions of it depending on their needs. The loan will be repaid with a 10% interest and 1-2% transaction fees over a period of 24 months. The exact payment plan will developed between the CMW and the MFI. Mercy Corps will provide a cash deposit to act as collateral for the loan component. Additionally, equipment worth $680 and CMWs Kit for Home Deliveries worth $116 will be provided to each participating CMW using grant funds. While Mercy Corps understands that the government may be unlikely to take up this grant component of the program, we could not place all of the set-up fees within the loan due to reservations from MFIs. Using resources from her loan, each CMW will dedicate and prepare a room with essential furniture (sheets, table, toilet, refrigerator, etc.).

3. Creating a demand for CMW services through a mass-media BCC campaign and women's groups. With support from Pak Vista Shared technologies, mobile phone will be used to transmit SMS messages to CMW clients and family members*.* The messages will be designed to raise community awareness about MNCH. Pak Vista’s software solution will adapt the open source software *CommCare*, developed by Dimagi and supported by USAID in multiple countries. When a client registers with a CMW, she will have the option to sign up for: (a) SMS reminders for her next visit and/or (b) voice-based health messages. Since literacy rates are low, the signed up clients will receive an SMS *image* that communicates the messages. The CMW will teach the client how to interpret the image. Mass VOIP (voice over internet protocol) calls will transmit pre-recorded health messages. Pak Vista Shared Technologies is currently exploring the feasibility of sharing messages tailored to the client's stage of pregnancy. Initially the program will pay for the calls, but the server will be transitioned to the Government of Baluchistan by the end of the project.

At the same time, the CMWs, together with the LHWs, will be actively engaged in awareness raising and demand-creation using Women Support Group (WSG) methodology. After receiving training from Mercy Corps, the CMWs and LHWs will develop joint work plans to establish four to five WSGs in each CMW catchment area, each of which will contain around 10 Lead Mothers. These Lead Mothers will then hold education sessions with 10-15 of their neighbors. In return for their services, CMWs will offer non-monetary incentives, such as free clinical services, to the Lead Mothers and their children.

The messages for the VOIP system and the WSGs will be developed using the *Designing for Behavior Change (DBC)* framework including formative research to identify enablers and barriers. This formative research will inform the entire communication strategy, including refinement of existing behavior change materials for the Women Support Groups, as well as the mass SMS campaign.

**Table 1:** CMW selection criteria

Required criteria^*^	Completed 18 month training, and passed National Examination Board
	Resident of catchment area
	Catchment population at least 3,000 (i.e. the CMW can reach communities/households in her catchment area within 1 hour travel distance, using transport available to the CMW)
	Willingness to complete the tasks of the CMW’s job description, including:
	o Work as skilled birth attendance
	o Participate in trainings
	o Repay loans
	Computerized National ID Card (CNIC) (Most CMWs should already have this, as it’s a requirement to register in CMW training course.)
	Local/domicile certificate
	Space available in home and willingness to utilize space for work station
	Only one CMW will be selected from a catchment area
	Mobile network coverage in home community
Preference will be given to	Married women
	Currently uses mobile phones
	Registered with Pakistan Nursing Council

*These criteria were developed in partnership with the government of Baluchistan to ensure that the project is most likely to succeed, while still serving the needs of vulnerable and marginalized communities.

#### Operations research

A key element of the SMNC initiative - the provision of business skills training and loans to establish private practices - is an innovative initiative in Pakistan and thus little is known about its effectiveness. A similar project was undertaken in Indonesia in 1993, but it only consisted of provision of loans [13]. Moreover, the selection criteria for loans ensured that only midwives with established practice could access them. While this was done to ensure repayment, it resulted in the exclusion of village midwives that form the pillar of the Indonesian midwifery program in rural areas. The outcome of interest was also limited to family planning clients. The scope of SMNC is broader; it includes, in addition to the loans, business skills training, clinical refresher training, supportive supervision and demand generation of CMW services using innovative technology. The proposed research, therefore, aims to answer the following questions:

## Research questions

1. Has the SMNC initiative had an impact on CMW service uptake? Has the initiative increased coverage of the full scope of skilled maternity and newborn care for women living in the interventions districts?

2. If there is an increase in CMW service uptake, is this increase attributable to the SMNC initiative?

3. Have the CMWs become satisfied, self-sustaining, quality private providers?

a) Has the business skills training enabled the CMWs to establish viable practices and provide high quality care?

b) Have the financial resources provided been helpful in allowing the CMWs to become self-sustaining? Are the loans being used as intended? How exactly are the loans enabling CMWs to establish their practices?

c) What barriers hinder CMWs from becoming self-sustaining private health care providers?

d) d) Has involvement in SMNC initiative improved the CMWs' living conditions and quality of life?

**Figure 1:**
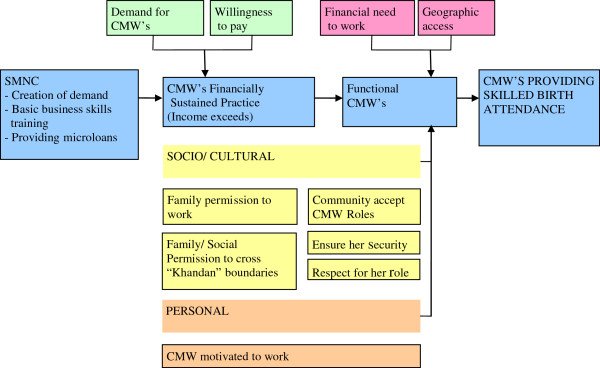
Conceptual framework of the SMNC operations research.

4 What is the quality of care the CMWs provide, both from perspectives of evidence-based best practice and of women receiving services? Figure 1.

## Study objectives

**Figure 2:**
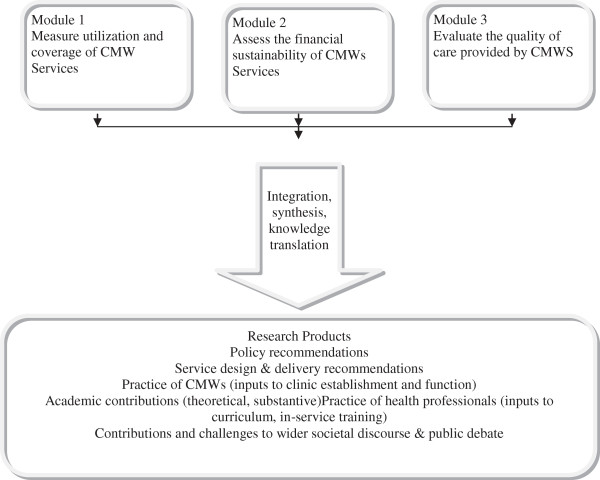
Diagrammatic representation of the research design.

The fundamental objectives of the research are:

1. To develop evidence that the SMNC initiative has led to increased coverage of high quality maternal and neonatal health care by trained, private-sector community midwives in underserved and insecure districts of Baluchistan.

2. To explore whether CMWs’ access to business skills training, small loans, and infrastructural support has enabled them to develop financially sustainable private midwifery and neonatal practices. To enhance empirical understanding of the ways in which these processes operate.

3. To map women’s experiences of maternal and neonatal health care provided by the CMWs, specifically their perceptions of the quality of care provided and ability to access to emergency maternal and neonatal care.

4. To inform positive developments in maternal health policy, service design and care delivery in Baluchistan, Pakistan more generally and elsewhere. Figure 2.

## Methods/Design

A mixed methods approach using both quantitative and qualitative methods will be used. Data will be collected in three overlapping modules over a 39-month period from districts Quetta and Gwader.

Module 1 will address research objective 1 and will consist of a quasi-experimental impact assessment.

Module 2 will address research objectives using a quantitative financial tool.

Module 3 will address research objective 3 to assess the quality of CMW services using a range of quantitative and qualitative inquiry methods.

## Methods and measurement of variables

### Module 1

Module 1 will consist of a cluster quasi-experimental impact assessment in districts Quetta and Gwader. Women receiving services in the catchment areas of SMNC-CMWs will be compared with women living in areas covered by a non-SMNC CMW, henceforth called the controls. This method will enable us to assess, with reasonable assurance that any changes in coverage of maternity and newborn care in SMNC-CMW catchment areas are *due to the SMNC intervention*. Data will be collected using a baseline and end-line survey within the context of cluster quasi-experimental methods.

#### Setting and population

The intervention groups will consist of the SMNC-CMWs and women living in their catchment areas. The control groups will consist of non-SMNC-CMWs catchment areas in Union Councils (UC) that are separated from SMNC-CMW Union Council by at least one Union Council. This is to reduce contamination of the controls, but ensure sufficient matching of socio-economic characteristics. Controls will also be matched, where possible, by year of CMW deployment. The target populations will be married women aged 15-49, who gave who have given birth in the three years prior to the survey. In the baseline survey, the study population will include women who have given birth in the three years prior to the survey. In the end-line survey, the study population will consist of women who gave between August 2013 and May 2016.

#### Sample size and sampling

**Figure 3:**
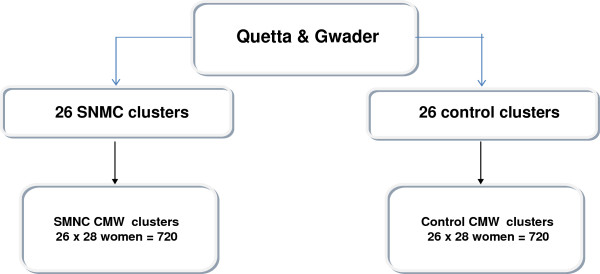
Quasi-experimental design sampling framework.

Sample size was calculated using the statistical package PASS 2012 [14]. program and is based on CMW coverage rates in district Layyah Punjab (3%). We estimate we require sample sizes of 760 women in group one (SMNC-CMW coverage areas) and 760 in group two (control-CMW coverage areas) to achieve 90% power to detect a difference between the group proportions of 0.0500 (i.e. to detect at least a 5% difference in coverage between the intervention and comparison areas). The proportion in group one (SMNC-CMW coverage districts) is assumed to be 0.05 under the null hypothesis and 0.1 under the alternative hypothesis. The proportion in group two (the control group) is 0.05. The test statistic used was the two-sided Z test with pooled variance. The significance level of the test was targeted at 0.05. Since cluster sampling will be done, this sample size takes into account a design factor of 1.3. A cluster in this research will consist of a CMW and her catchment area. The sampling frame will be drawn up using a two stage cluster sample design (see Figure 2). To interview 760 women in each group (total 1520 women), we will recruit all 26 CMWS in SNMC intervention and 26 control CMWs (52 CMWs total). The control CMWs will be randomly selected from CMW provincial database. Within each selected cluster, 28 women who gave birth in the three years prior to the survey will be interviewed [15]. The first house to be contacted will be randomly selected from the LHW registers. Every second house will be visited. We estimate that at least 124 households per cluster, who gave birth in the 3 years prior to the survey, will need to be *contacted*. This is based on a demographic assumption that 60% of households will have a woman aged 15-49, 30% of whom gave birth in the last three years [14] and a participation refusal rate of 15% (all conservative estimates) (DHS 2007). A survey of the selected CMWs will also be done to capture their socio-demographic data. Figure 3.

#### Data collection

Data will be collected using modified MCHIP survey questionnaires (Rapid Knowledge, Practices and Coverage (KPC) survey module 5: Maternal and Newborn care (2008)). The tool will contain measures of the full range of essential maternal and neonatal health care the CMWs are expected to provide - ANC care, skilled birth attendance, maternal tetanus toxoid (TT) vaccination, postnatal care, modern contraceptive use, cord care, thermal control, early initiation of breastfeeding and exclusive breastfeeding. Additional questions will allow us to capture socially excluded groups and women's satisfaction with CMW care. Information will also be collected around the financial costs of seeking CMW care, and other social and geographic barriers to seeking her care. If a woman cannot be interviewed at the time of contact, a mutually agreed upon time and place will be scheduled for another visit and interview. Women will be interviewed by female enumerators. It is anticipated that most interviews will last approximately 45 to 60 minutes. A separate survey questionnaire will be used to capture CMW socio-demographic data.

#### Data analysis

Data will be analyzed using Stata 12.0, using the program’s ‘svy’ set of commands that take into account the design effect of clustering and stratification [16]. Univariate and bivariate analyses will be done to assess the proportions of births that were provided a full range of essential maternal and newborn care by the CMWs at baseline and endline. Logistic regression models will then be developed to estimate the odds of birth attendance by CMW in the SMNC districts compared to control districts, controlled for potential confounders. Are women in SMNC districts more or less likely to use a CMW compared to women living in control districts? How satisfied are women with the care provided by SMNC CMWs compared to control CMWs? Is there any variability in birth outcomes between the two groups of women under the care of a CMW?

#### Personnel

A core team of 8 data collectors (7 women and 1 man) will be trained. The hiring and training of more women than men is necessary as female interviewers will be the primary data collectors. The male data collectors will interview the husbands or other men if the women are unable to answer all the questions. All enumerators will be trained in the technical details of survey data collection and in cultural sensitivities of the local context (how to approach people, their mannerisms and language, that their personal attire should align with the local cultural expectations and not be offensive). The research manager will oversee the entire survey, ensuring correct coverage and quality of the data. A quality-monitoring check list will be used to ensure the quality of data collected. The Principal Investigator (PI) will review the work on a daily basis and monitor data quality weekly. Completed questionnaires will be edited and entered into Stata 12.0 using a double-entry system.

### Module 2

Module 2 will address research objective 2. It will explore the financial sustainability of the SMNC-CMWs’ midwifery practice. Specifically, we will assess: (1) if the SMNC initiative of providing the CMWs with business skills training and micro-credit loans has enabled the CMWs to establish self-sustaining practices and (2) if involvement in the SMNC initiative has improved the quality of their lives as they perceive it. A quantitative financial tool will be used to assess the direct and indirect monetary costs as well as opportunity costs and benefits. These costs include financial requirement of establishing practices and maintaining equipment. In addition, we may evaluate the temporal and monetary costs of organizing chaperones (i.e. depending on female family members to accompany young CMWs to deliveries during the day and two or more male chaperones for night deliveries) and private transportation fees.

#### Data collection and study population

Data for financial sustainability of CMW practices will be collected using a pre-tested financial tool. Program Records will also be used. Twenty SMNC-CMWs (10 in each district Quetta and Gwader), will be randomly selected from the 50 CMWs who have agreed to participate in the SMNC initiative in Year 1. They will be interviewed in-depth and asked to fill out a pre-tested questionnaire at three intervals of time; baseline, 16-month mark and 36-month mark. Direct or indirect costs shall be calculated on market rates.

#### Personnel

A woman trained in financial analysis (Masters Economics) will collect and analyze the financial data. In-depth interviews with CMWs will be conducted by an experienced Anthropologist (Master’s degree holder, woman).

#### Data analysis

To assess financial sustainability of CMW practices, their direct, indirect and opportunity costs will be calculated using Microsoft Excel. An income and expenditure statement will be developed for each CMW. The qualitative data (from in-depth interviews) will be analyzed using Atlas Ti [17]. Since the data will be collected in Balochi and Pushto, data will first be translated and transcribed by native speakers. A database of the transcribed interviews and focus group discussions will be created. Using a social constructivist, interpretative approach [18], data will be coded and broad themes identified. Initial coding will be guided by the stated research objectives and later by additional concepts as they emerge. Data analysis will be an on-going and iterative process through all phases of data collection, as early identification will allow a fuller probing of unanticipated concepts and variables in upcoming interviews and focus group discussions [19]. Interpretive accuracy will be assessed by triangulation of findings across the four phases, peer debriefing within the research team and other colleagues, and respondent validation.

### Module 3

Module 3 will address research objective 3. It will assess the quality of CMW services by: (1) mapping CMW care against clinical evidence-based best-practices and (2) women’s experiences of maternity and newborn care, specifically skilled attendance at birth in both normal and emergency obstetrical scenarios. Their perspectives of what constitutes high quality CMW care will be mapped.

#### Methods

Drawing upon the Hulton, Mathews & Stones [20] framework for assessing quality of maternal health services in developing countries, the CMW quality of care assessment will include an assessment of their practice's physical and instrumental infrastructure and their knowledge and skills to attend a normal birth, identify birth complications and provide essential newborn care. The quality of their referral links, information management systems and the use of appropriate technologies in caring for women and newborns will also be assessed.

Women’s experiences of maternity care will include an assessment of their impression of the adequacy of CMWs’ human and physical resources, their trust in her competency to provide safe birth attendance, identify birth complications (or potential for complications), manage an emergency (both maternal ad newborn) and provide the necessary referral services. The respect they are accorded, their sense of dignity, equity, and the emotional support they receive from the CMW will also be assessed.

#### Data collection

Specifically, the following will be done:

1. Observation and documentation of 12 CMW birth facilities, both physical (e.g., whether the design of the labour room respects women’s privacy) and instrumental (e.g., Availability of instruments, their quality, availability of essential drugs and supplies) using a structured checklist [21].

2. Observation of 25 moment-in-time CMW-patient interactions*.* This will include an observation of at least five births overall*.* The points of observation will include, but not be limited to, the provider’s language and behavior toward the patient and her family, time spent with each patient and whether her concerns are addressed. Any complications or adverse outcomes will be explored in depth, which will include interviewing the woman, her family and the CMW.

3. Exit interviews with ten women who delivered in CMW practices (5 routine deliveries and 5 who required emergency obstetric care; 10 interviews total).

4. *In*-depth interviews with 20 CMWS and 5 referral physicians, both in the public and private sector*.*

#### Personnel

The research team for Modules 3 will be comprised of an experienced Anthropologist (Master’s degree holder, woman) and one research assistant (Anthropologist – Master’s degree holder).

#### Data analysis

Data will be analyzed as described above in 5.2.3.

## Ethical considerations

Ethics approval has been obtained from the University of Alberta Health Research Ethics Board and Pakistan Bioethics Board [22].

Maintaining privacy and confidentiality of all the study participants and is an important ethical issue. This will be addressed by only collecting the necessary information [23]. Only the researchers and project manager will have access to the list of participants’ names and associated codes. An equally important ethical concern is the security of the research team. A separatist movement in Baluchistan has created a fragile security situation. Efforts to ensure security of the research team include 1) Following Mercy Corps security and travel advice 2) Hiring local staff familiar with the terrain and social context 3) renting low-profile, unmarked vehicles; and 3) Following common-sense precautions like avoiding night travel.

## Discussion

The goal of this research is to document the impact of an intervention aimed at enabling Community Midwives to establish financially sustainable private practices while providing services to women and children living in remote rural areas. Two unique elements of the intervention are provision of loans to community midwives and use of m-technology to increase demand for biomedical maternal health services. Given the dominance of the private sector in delivery of maternal health services and emerging importance of m-health in creation of demand for services, this research is very timely as it will provide empiric evidence of the feasibility of these ideas in a conservative, remote rural population beset with militant activity. A key strength of our study is the use of a quasi-experimental research methodology to assess the impact of the SMNC intervention. Comparison of intervention cases with controls will allow us to conclude, with some validity, whether or not any changes in CMW coverage and quality of care can be attributed to the SMNC initiative. Another strength of our research is use of multiple mixed methods research.

## Competing interests

The authors declare that they have no competing interests.

## Authors’ contributions

ZM conceptualized the research project. AC developed the SNMC intervention. All authors have approved the final version of the manuscript.

## Authors’ information

ZM (MBBS, MPH, PhD (Public Health Medicine) (PI), is a medical doctor, with expertise in gender and reproductive health with a particular focus on women’s access to reproductive health services and inequities in reproductive health policy, design and delivery of services.

AC (MPH) is the Senior Technical Health Advisor, Mercycorps, Pakistan.

AB (MBA) is the Research Manager, Real Medicine Foundation and is responsible for implementation of the research activities.

## Pre-publication history

The pre-publication history for this paper can be accessed here:

http://www.biomedcentral.com/1471-2393/14/131/prepub
